# The radiodensity of cerebrospinal fluid and vitreous humor as indicator of the time since death

**DOI:** 10.1007/s12024-016-9778-9

**Published:** 2016-04-27

**Authors:** Desirée H. J. L. M. Koopmanschap, Alireza R. Bayat, Bela Kubat, Henri M. de Bakker, Mathias W. M. Prokop, Willemijn M. Klein

**Affiliations:** 1 Department of Radiology and Nuclear Medicine, Radboud University Medical Center, Geert Grooteplein Zuid 25, 6525 GA Nijmegen, The Netherlands; 2 Department of Pathology, Netherlands Forensic Institute, Laan van Ypenburg 6, 2497 GB Den Haag, The Netherlands; 3 Department of Pathology, Maastricht University Medical Center, P. Debyelaan 25, 6229 HX Maastricht, The Netherlands; 4 Department of Radiology, Groene Hart Ziekenhuis, Bleulandweg 10, 2803 HH Gouda, The Netherlands

**Keywords:** Postmortem computed tomography, Radiodensity, Cerebrospinal fluid, Vitreous humor, Postmortem interval

## Abstract

**Purpose:**

After death, a series of changes occur naturally in the human body in a fairly regular pattern. These postmortem changes are detectable on postmortem CT scans (PMCT) and may be useful in estimating the postmortem interval (PMI). The purpose of our study is to correlate the PMCT radiodensities of the cerebrospinal fluid (CSF) and vitreous humor (VH) to the PMI.

**Methods:**

Three patient groups were included: group A consisted of 5 donated cadavers, group B, 100 in-hospital deceased patients, and group C, 12 out-of-hospital forensic cadavers. Group A were scanned every hour for a maximum of 36 h postmortem, and the tympanic temperature was measured prior to each scan. Groups B and C were scanned once after death (PMI range 0.2–63.8 h). Radiodensities of the VH and CSF were measured in Hounsfield units. Correlation between density and PMI was determined using linear regression and the influence of temperature was assessed by a multivariate regression model. Results from group A were validated in groups B and C.

**Results:**

Group A showed increasing radiodensity of the CSF and VH over time (*r*^2^ CSF, 0.65). PMI overruled the influence of temperature (*r* = 0.99 and *p* = 0.000). Groups B and C showed more diversity, with CSF and VH radiodensities below the mean regression line of Group A. The formula of this upper limit indicated the maximum PMI and was correct for >95 % of the cadavers.

**Conclusion:**

The results of group A showed a significant correlation between CSF radiodensity and PMI. The radiodensities in groups B and C were higher than in group A, therefore the maximum PMI can be estimated with the upper 95 % confidence interval of the correlation line of group A.

## Introduction

Estimating the time of death is an important part of forensic pathology with the postmortem interval (PMI) being defined as the period of time that has elapsed since a person has died. In a medico-legal investigation of death, an accurate estimation of the PMI can be of great importance. It may aid in identification of the victim and, in cases of a violent death, limit the number of suspects and help to validate or reject an alibi and verify witnesses’ statements [1]. Also, in the postmortem diagnostic follow-up in a clinical setting, knowledge about the PMI is pertinent in being able to differentiate between normal postmortem changes and pre- or peri-mortem pathology [2–4].

Several techniques can be used if the time of death is unknown. In the immediate hours after death, medical examiners and pathologists usually ascertain the PMI utilizing early postmortem changes such as algor mortis (the decrease in body temperature after death), livor mortis (postmortem lividity or hypostasis), and rigor mortis (chemical change in muscle tissue that causes stiffening) [5]. These traditional methods have been accompanied by numerous tests or rules-of-thumb such as the drop in body temperature, which is used in the Henssge nomogram in combination with body weight and clothing, resulting in a more precise estimation of PMI [5, 6]. Biochemical measurements such as creatine levels in cerebrospinal fluid (CSF) and vitreous humor (VH) have been investigated, as well as CSF pleocytosis and trace amines [7–11]. These markers show a good correlation to the PMI, however, the wide range of estimated times of death makes them less accurate than the traditional triad of algor, livor, and rigor mortis.

For around a decade, postmortem computed tomography (PMCT) and postmortem magnetic resonance imaging (PMMRI) have been introduced into clinical and forensic postmortem investigations, to visualize disease and cause of death and to serve as a virtual guide for autopsy [12, 13]. It is interesting to test the possibilities of PMCT in aiding the estimation of time of death as these digital scans may contain more information than just morphology. The vitreous humor and cerebrospinal fluid may show the least transformation in the postmortem body, as these are relatively closed compartments, normally without an open access to the exterior and usually devoid of bacteria. The purpose of our study is to correlate the PMCT radiodensities of the CSF and VH to the PMI.

## Materials and methods

### Study population

The correlation between PMI and radiodensities of CSF and VH was determined in 3 distinct groups of cadavers, with Group A as the standard and Groups B and C validation groups. In all groups PMCT included head, cervical spine and a thoracic-abdominal series. For this study cadavers were scanned in a natural, supine position with arms next to or on the torso, using the scan parameters as shown in Table 1. No intravascular contrast agent was administered, images were ordered caudal to cranial and a soft filter was applied for the cerebral images.

**Table 1:** CT-scanners and scan parameters

Type scanner, detector row	Siemens 16^a^	Siemens 64^b^	Aquilion 320^c^	Aquilion 64^d^
Detector collimation (mm)	16 × 0.75	16 × 0.75	16 × 0.75	64 × 0.5
Reconstruction interval (mm)	0.75	0.75	0.75	0.5
Tube voltage (kV)	120	120	120	120
Tube current (mA)	400	400	400	350
Rotation time (s)	1	1	1	1

^a^Siemens Somatom Sensation16, Siemens Healthcare, Germany

^b^Siemens Sensation 64, Siemens Healthcare, Germany

^c^Aquilion ONE 320, Toshiba Medical Systems Nasu, Japan

^d^Aquilion 64, Toshiba Medical Systems Nasu, Japan

### Group A: donated cadavers

Individuals who had donated their bodies for scientific research after their death were prospectively included for PMCT scanning from January to March 2014. Ethical approval was waived as these individuals had already consented to any scientific procedure. This is also in accordance with Dutch law. Date of birth, sex, cause, and time of death were provided by the undertaker and the cadavers quickly transported, without a cooling period, to a dedicated CT suite. Immediately after arrival the cadavers were scanned using a 16-slice scanner (Siemens Somatom Sensation scanner, Siemens Healthcare, Germany), every hour for up to a maximum of 36 h postmortem. During this time the cadavers were not moved and prior to each scan, tympanic temperature, using a Basetech LT-80 Digital thermometer, was measured in both ears and the mean calculated. The temperature within the CT suite was 22 to 25 °C with an air humidity varying from 24 to 57 % (TFA Thermo-/hygrometer).

### Group B: in-hospital cadavers

Patients who had died in our university hospital or in our emergency room and had had a cerebral PMCT scan, were prospectively included in this group from July 2012 to January 2015. Exclusion criteria were: intracranial intervention or hemorrhage for the CSF measurements and after ocular donation for the VH measurements. Relatives were required to give consent for the PMCT and the local ethical committee waived any ethical approval for study purposes. Basic patient data, including time and assumed cause of death, were obtained from the digital patient files. The PMCT was performed as soon as possible after death and whenever possible, prior to an eventual autopsy. If immediate scanning was not possible, the cadaver was stored in the mortuary at 4 °C. The scanners used were, Siemens Somatom Sensation 16, a Siemens Sensation 64 (Siemens Healthcare, Germany) or an Aquilion ONE (Toshiba Medical Systems, Japan). Temperatures of these cadavers were not measured with the scan suite temperature and humidity standardized according to the hospital settings.

### Group C: out-of-hospital cadavers

Out-of-hospital cadavers from a forensic institute were retrospectively included if the head anatomy was intact (without fractures, wounds, or corpora aliena). Patient data included the cause and the assumed or estimated time of death, based on forensic investigations. The cadaver was transported to the CT suite after forensic investigation of the crime scene and having being stored in the mortuary. The PMCT was performed with the body in a natural supine position, using an Aquilion 64 (Toshiba Medical Systems, Japan). The temperature of the cadavers was not measured in the CT suite. During the scan procedure, room temperature and humidity were standardized according to the institutes’ settings.

### Radiodensity measurements

**Fig. 1:**
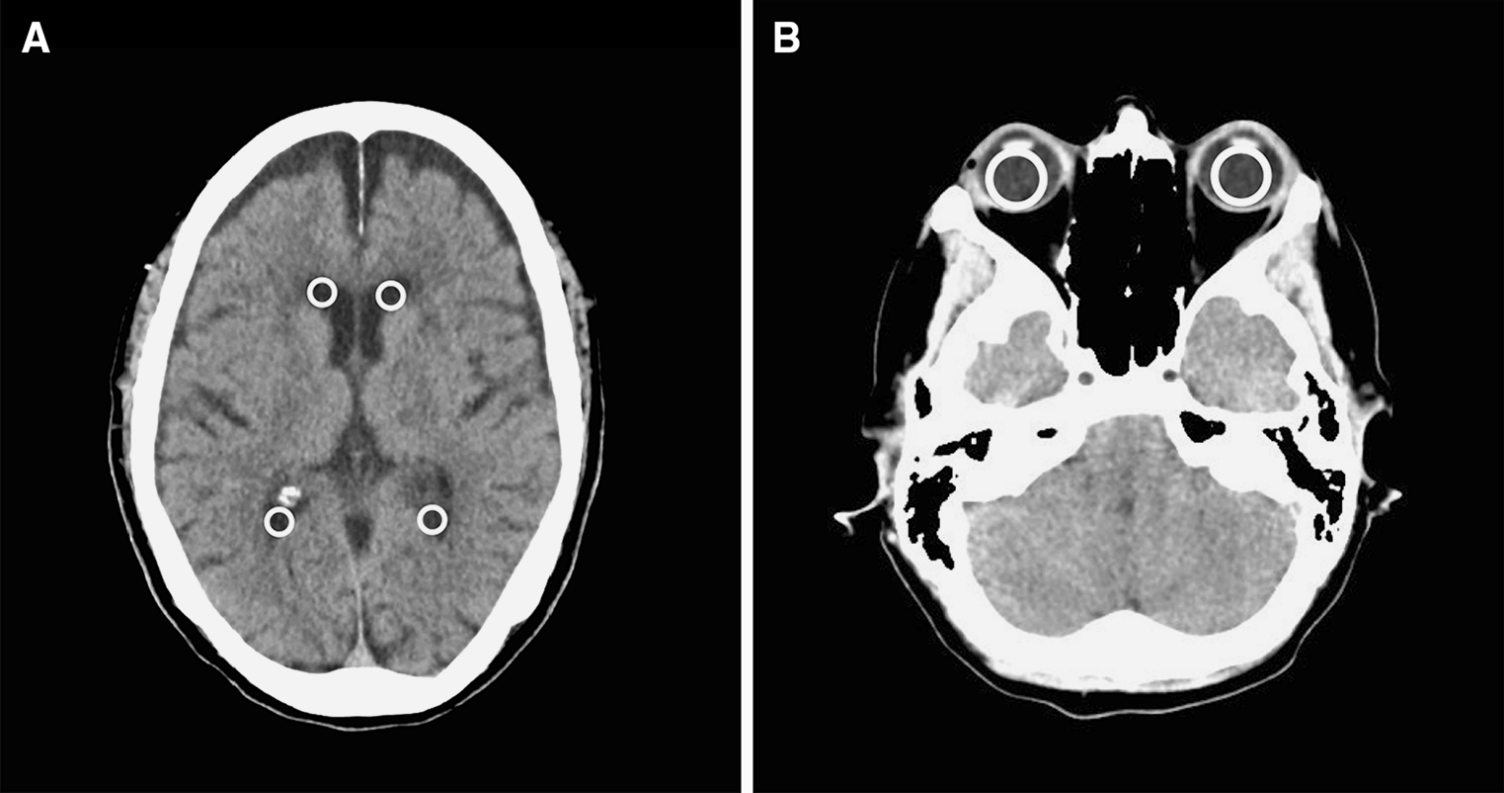
The radiodensity, in Hounsfield units (HU), was determined by the mean values in the maximum fitting circular regions of interest (ROI). ROI’s were positioned in the anterior and posterior horns of the lateral region of the *left* and *right* ventricles (**a**) and in the vitreous humor of both eyes (**b**)

Radiodensity in Hounsfield units (HU) of the VH and CSF were measured by two researchers. Circular regions of interest (ROI) automatically calculated the mean using IMPAX (software version 6.5.3.1005, Agfa Healthcare). The radiodensity of the CSF was determined by the mean of 4 measurements with a maximum fitting ROI positioned in the anterior and posterior horns of the left and right ventricles, excluding the choroid plexus or visible beam hardening artifacts of plexus calcifications. The radiodensity of the VH was determined by the mean value of the left and right VH, excluding the lenses (Fig. 1).

### Statistical methods

Data was analyzed using SPSS (IBM Corp. Released 2011. IBM SPSS Statistics for Windows, Version 20.0. Armonk, NY: IBM Corp).

The correlation between the PMI and postmortem CSF and VH density measurements were evaluated using linear regression. In Group A, a mixed model was used to predict the PMI, in hours, with the fixed effect being the mean radiodensity. A random intercept and slope was calculated per cadaver to manage the multiple observations per individual. The fixed effect regression line is the average regression line of the donated cadavers. Using the fixed effect regression line, a prediction of the maximum PMI was calculated to validate this model for Groups B and C and was compared to the actual PMI. Results are shown in scatterplots [14].

Correlations of the temperature and the PMI to the radiodensity of CSF and VH were analyzed in multivariate regression models. The inter- and intra-observer reliability were calculated with interclass correlation (ICC) and 95 % confidence interval (CI), using a random sample of 15 cases in group B [15]. Pooled standard deviation, using one-way ANOVA, was determined to estimate the random error of the density measurements of the CSF and VH, between and within observers respectively.

## Results

### Study population

Five donated cadavers, 100 in-hospital deceased patients and 12 out-of-hospital forensic cadavers were included in this study. Basic patient data are shown in Table 2.

**Table 2:** Basic patient data

Patient data	Group A Donated cadavers	Group B In-hospital cadavers	Group C Forensic cadavers
Number	5	100	12
Gender			
M:F (%: %)	4:1 (80:20 %)	62:38 (62:38 %)	9:3 (75:25 %)
Age (years)	77.5	58.0	48.4
Mean (SD, range)	(9.0, 68–90)	(21.2, 0–91)	(14.5, 29–84)
Referring department/location of death			
Hospital ward	1	36	
Intensive care		16	
Emergency room		32	
Out-of-hospital		15	12
Unknown	4	1	
Cause of death			
Neoplasm	2	5	0
Exsanguination	0	21	5
Ischemia	0	14	3
Infection	0	29	1
Accidental and non-accidental injury	0	1	2
Systemic disease	0	9	2
Perforation/obstruction	0	8	0
Undetermined	3	13	0
Postmortem interval (h) mean (SD, range)	First scan: 7.6 h (1.7, 6–10 h)	12.6 h (11.8, 0.2–63.8 h)	22.3 h (8.0, 15.0–42.3 h) (estimated)

N.B. the postmortem interval of group C comprising forensic cadavers, is based on the estimated time of death after forensic investigations and autopsy

Correlation coefficient of the radiodensities with the PMI of each cadaver in group A

#### Group A

This group included 4 males (80 %) and 1 female (20 %) donated cadavers, with an age range from 68 to 90 years (mean 78.8 years). The PMI of the first scan varied from 6 to 10 h, with a mean PMI starting at 7.6 h. Measurements of CSF and VH radiodensity on the PMCTs were performed in all cases. Tympanic temperature was measured in only 4 of the 5 cadavers due to a technical failure.

#### Group B

The in-hospital cadavers consisted of 62 males (62 %) and 38 females (38 %). Their age ranged from 0 to 91 years (mean 58.0, SD 21.8) and the PMI at PMCT ranged from 0.18 to 63.8 h (mean 12.6, SD 11.8). VH density measurements were performed in 99 cadavers as 1 cadaver was excluded due to a cornea donation prior to the scan. Another cadaver had an ocular prosthesis of the left eye so only the right eye was included. Density measurements of the CSF were performed in 98 of the 100 cadavers as one cadaver was excluded due to intracranial hemorrhage and in another cadaver, measurements were not possible as the ventricles had collapsed.

#### Group C

The forensic cadaver group comprised 9 males (75 %) and 3 females (25 %) with an age range of 29–84 years (mean 48.4, SD 14.5). Four individuals had died after gunshot wounds to the thoracic-abdominal region, but without any intracranial wounds. Two individuals died of asphyxia (1 carbon monoxide intoxication, 1 strangulation), two other individuals died after blunt trauma and one after a fall from height. Three individuals were found with an unknown cause of death and after forensic investigations, cause of death was determined to be due to natural causes (1 myocardial infarction and 2 infectious diseases). Measurements of CSF and VH radiodensity were performed in all cases.

### Cerebrospinal fluid

The radiodensity of CSF on the PMCT of group A, showed an increase over time for all 5 cadavers, with radiodensities of 5.7–8.1 HU on the first scan at a PMI of 6–10 h, followed by an almost linear increase over time to a radiodensity of 10–12 HU at 36 h.

**Fig. 2:**
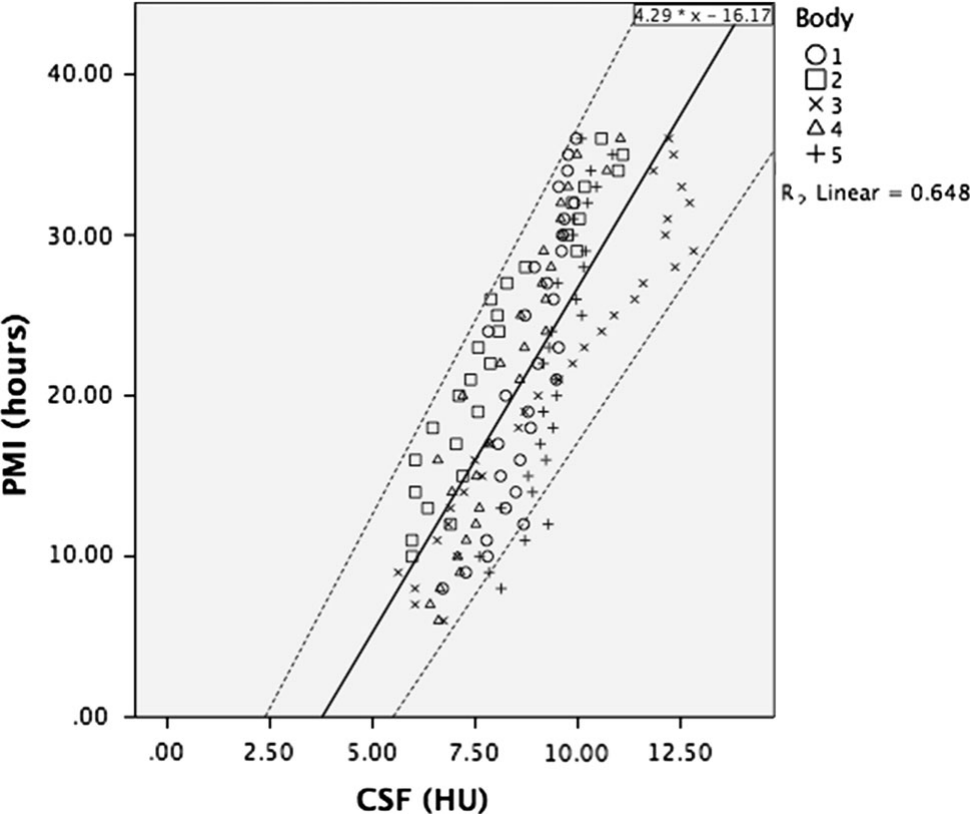
Scatterplot of the radiodensities of the CSF in the 5 donated cadavers that were scanned hourly, starting at a PMI of 6–10, to 36 h postmortem. *R* ^2^ is 0.65, which indicates a fairly accurate application of the model. The middle continuous line denotes the linear correlation line fitting the formula PMI (*h*) = 4.29 × HU^CSF^ − 16.17 and the *upper* and *lower lines* denote the 95 % confidence interval

The correlation of the radiodensity of the CSF with the PMI of each individual cadaver is shown in Table 2, showing high correlations for all 5 individuals. The fixed effect regression coefficient (adjusted *r*^2^) for the CSF was 0.65, which indicated a fairly accurate application of the model (Fig. 2). The prediction formula based on this fixed effect regression analysis, using the intraclass correlation (SPSS Statistics), is:

$$ {\text{PMI}}\left( h \right) = 4.29 \, \times {\text{ HU}}^{\text{CSF}} {-}16.17 $$

To assess minimum and maximum PMI, the lower and upper limits of the 95 % confidence interval of the regression analysis were derived with the upper and lower limit parameters.

$$ {\text{PMI}}\;{ \hbox{max} } = 4.82 \, \times {\text{ HU}}^{\text{CSF}} {-} \, 11.47 $$

$$ {\text{PMI}}\;{ \hbox{min} } = 3.77 \times {\text{HU}}^{\text{CSF}} {-}20.88 $$

**Fig. 3:**
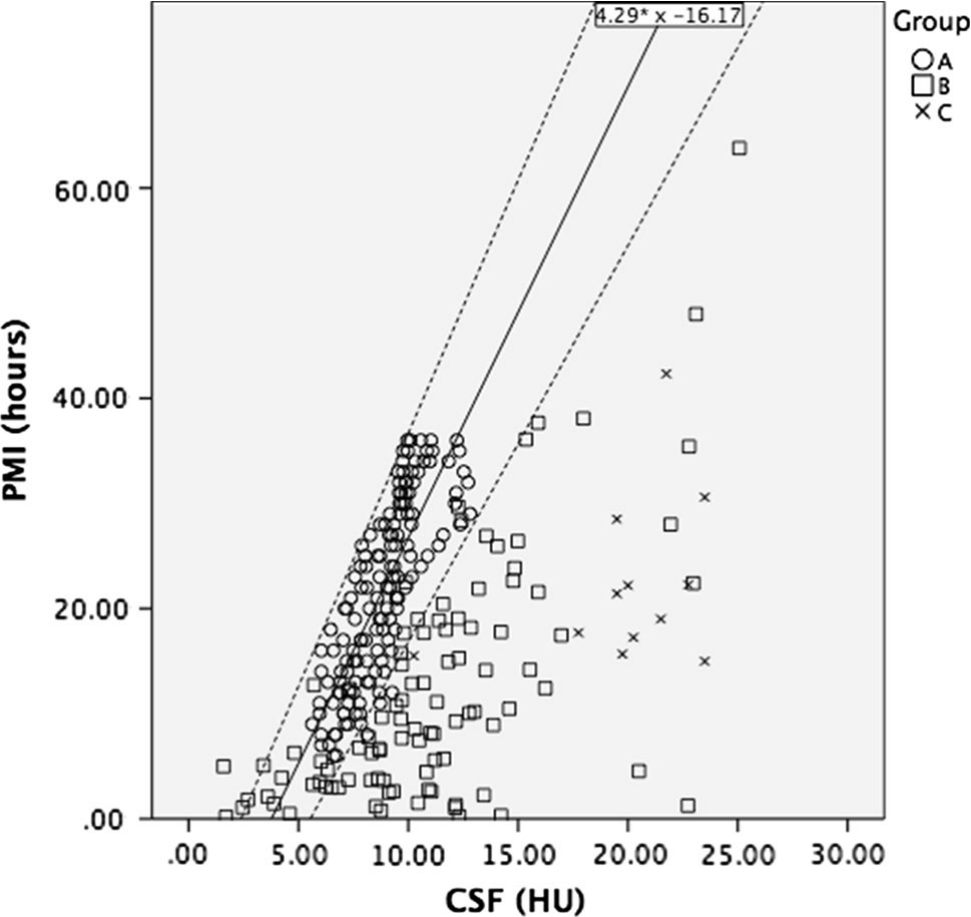
Scatterplot showing the CSF radiodensities of patients in groups B and C. The middle *continuous line* denotes the correlation line of the CSF radiodensity of group A, fitting the formula 4.29 × HU^CSF^ − 16.17. *Dashed lines* denote 95 % confidence interval

**Fig. 4:**
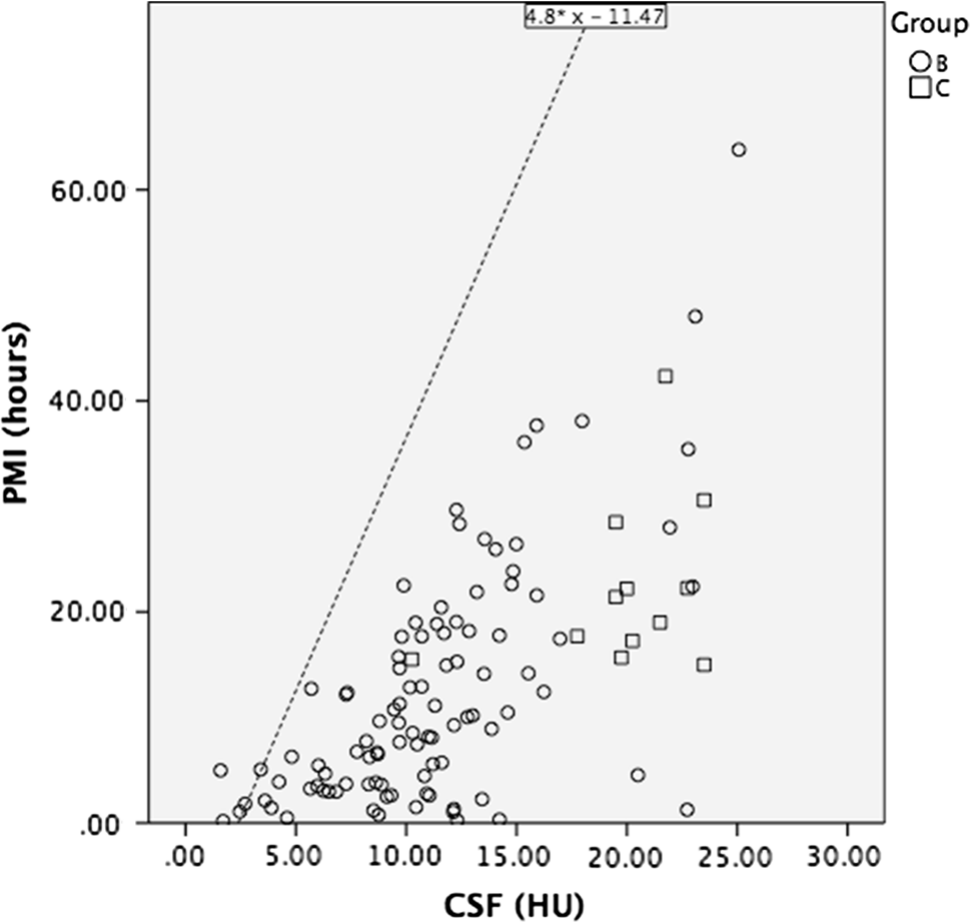
Scatterplot showing the CSF radiodensities of patients in groups B and C. The line denotes the upper limit prediction of the CSF radiodensity of group A, corresponding to the formula 4.82 × HU^CSF^ − 11.47, indicating estimated maximum postmortem interval

The measured radiodensities of the CSF of groups B and C are plotted against PMI in Fig. 3. The CSF radiodensities in groups B and C increase with the PMI, however, the correlation is poor. The CSF radiodensity was higher than predicted in the model of group A, not lower, therefore the upper limits of the 95 % CI obtained from group A can be used to estimate the maximum PMI. The PMI was at, or below the estimated maximum PMI in 93 of the 98 (95 %) cadavers in group B and in 12 out of the 12 (100 %) cadavers in group C (Fig. 4).

### Vitreous humor

**Fig. 5:**
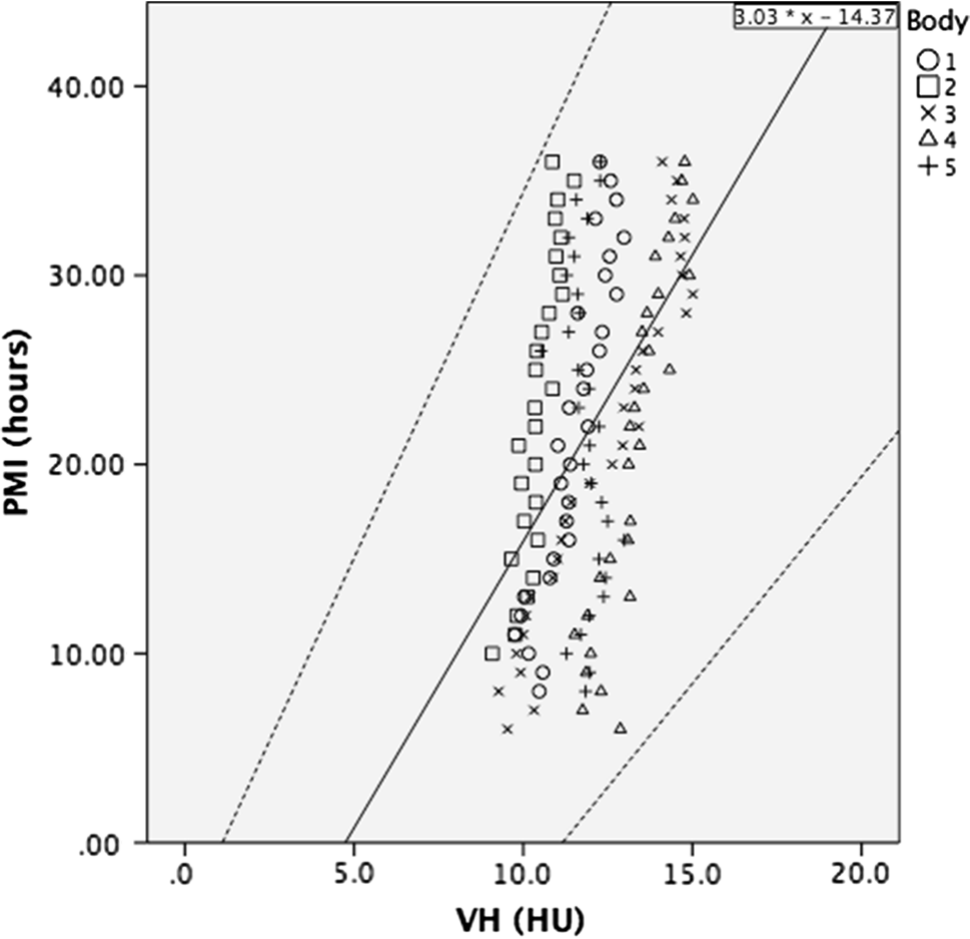
Scatterplot of the radiodensities of the VH in the 5 donated cadavers that were scanned hourly, up to 36 h postmortem, starting at a PMI of 6–10 h. The fixed effect regression line *r* ^2^ is 0.26, which indicates a low correlation. The *middle continuous line*, denotes the linear correlation line corresponding to the formula PMI (*h*) = 3.03 × HU^VH^ − 14.37 and the *upper* and *lower lines* indicate the 95 % confidence interval

The radiodensity of VH of group A tended to increase over time for all 5 cadavers, with radiodensities of 9.1–12.9 HU on the first scan at a PMI of 6–10 h. The individual plotted lines on the scatter graphs showed a slight increase in radiodensity during the ongoing PMI. The individual correlation in *r*^2^ is shown in Table 3. The fixed effect regression line, has a *r*^2^ of 0.26 (Fig. 5). The prediction formula based on this fixed effect regression analysis is:

$$ {\text{PMI}}\left( h \right) = 3.03 \, \times {\text{HU}}^{\text{VH}} { - }14.37 $$

**Table 3:** The correlation (*r* ^2^) of the radiodensity of the CSF and VH with the PMI of each individual cadaver, was generally very good

	Body 1	Body 2	Body 3	Body 4	Body 5	Fixed effect
*r* ^2^ CSF	0.74	0.91	0.94	0.90	0.83	0.65
*r* ^2^ VH	0.84	0.77	0.93	0.84	0.08	0.26

The adjusted *r* ^2^ was fairly good for CSF and moderate for the VH radiodensity

Radiodensities in correlation with tympanic temperature and the postmortem interval

To assess minimum and maximum PMI, the lower and upper limits of the 95 % confidence interval of the regression analysis were calculated.

$$ {\text{PMI}}\;{ \hbox{max} } = 3.87 \, \times {\text{ HU}}^{\text{VH}} {-} \, 4.30 $$

$$ {\text{PMI}}\;{ \hbox{min} } = 2.19 \, \times {\text{ HU}}^{\text{VH}} {-} \, 24.44 $$

**Fig. 6:**
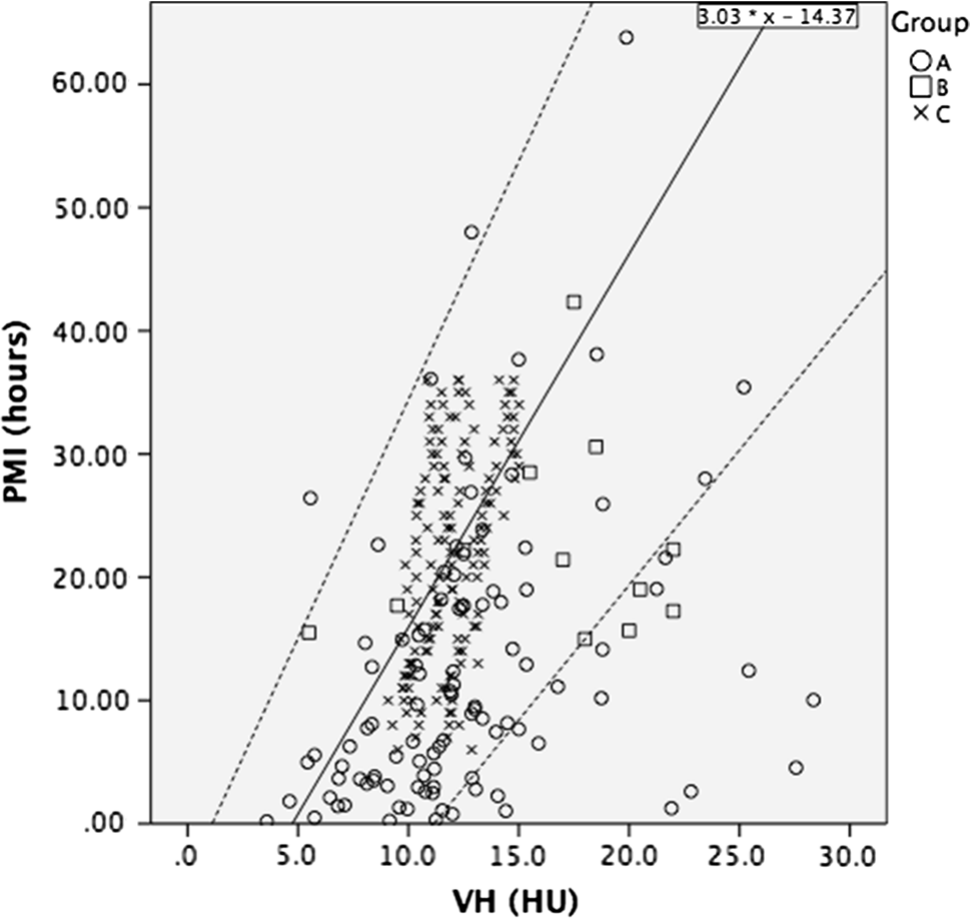
Scatterplot showing the VH radiodensities of patients in groups B and C. The *middle continuous line* denotes the correlation of the VH radiodensity of group A, corresponding to the formula 3.03 × HU^VH^ − 14.37. *Dashed lines* indicate 95 % confidence interval

**Fig. 7:**
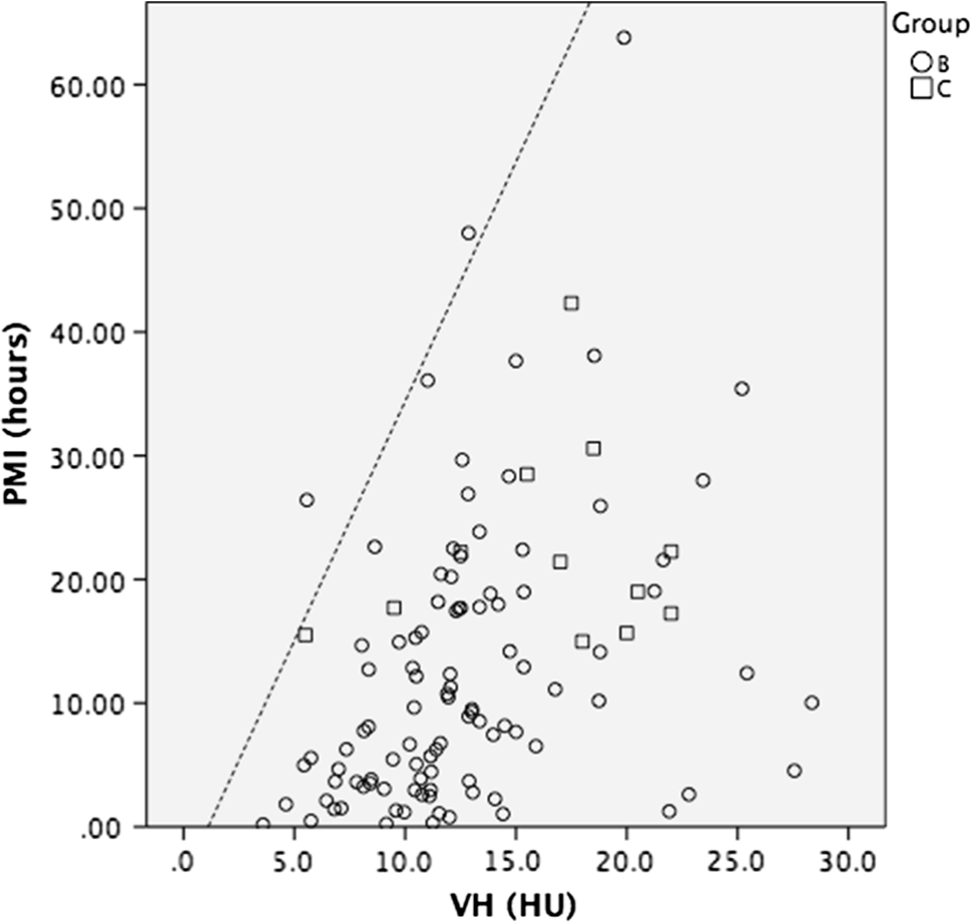
Scatterplot showing the VH radiodensities of patients in groups B and C. The *line* denotes the upper limit prediction of the CSF radiodensity of group A, corresponding to the formula 3.87 × HU^VH^ − 4.30, indicating the estimated maximum postmortem interval

The measured radiodensities of the VH in groups B and C also increased with ongoing PMI but showed even less correlation (Fig. 6). VH tended to have a higher radiodensity at the same PMI in groups B and C than expected from the prediction model generated from group A. Therefore, the formula of the regression line of group A cannot be used to estimate the PMI. The upper limit of the 95 % CI obtained from group A is exceeded in some cases (Fig. 7) and can be used to estimate the maximum PMI. The PMI was at, or below the maximum PMI in 96 of the 98 (98 %) cadavers in group B and in 12 of the 12 (100 %) cadavers in group C.

### Temperature

Tympanic temperature was measured prior to every scan in 3 cadavers, on both the left and right side, and in 1 cadaver, only on the left side. Temperature measurements of the first scanned cadaver was not measured due to a technical failure. The temperature showed a decrease from a mean of 31.4 °C at a PMI of 6 h, to a mean of 24.9 °C at a PMI of 36 h, consistent with the mean room temperature.

In univariate models, both tympanic temperature and PMI showed significant correlation to the radiodensity of CSF and VH. With both determinants in one multivariate regression model, PMI remained significantly correlated to the radiodensity of CSF and VH, whereas temperature measurements did not (Table 4).

**Table 4:** In a univariate model, both tympanic temperature and PMI showed significant correlation to the radiodensity of CSF and VH

	Beta	*p* value	*r* value
CSF radiodensity			
Postmortem interval (h)	0.99	0.00	0.99
Tympanic temperature (°C)	0.004	0.95	
VH radiodensity			
Postmortem interval (h)	1.13	0.00	0.96
Tympanic temperature (°C)	0.19	0.13	

With both determinants in one multivariate regression model, PMI remained significantly correlated to the radiodensity of CSF and VH, whereas temperature did not

### Intra- and inter-observer reliability

The intra-observer variability for the radiodensity measurements of left and right anterior and posterior CSF, varied from 0.82 to 0.94 and the inter-observer variability for CSF varied from 0.84 to 0.99. For VH, these results were, respectively, 0.89–0.94 for intra- and 0.83–0.90 for inter-observer variability in the left and right VH, indicating a very high reliability.

## Discussion

In this study radiodensities of CSF and VH increased after death in correlation to the PMI. In the multivariate model, PMI was a significant predictor of CSF radiodensity whereas temperature was not. We calculated a predictive formula to estimate the maximum PMI with the radiodensity of CSF.

Worldwide, the use of postmortem radiology is expanding in both forensic and clinical postmortem diagnostic work-up, which is partly in response to the decreasing numbers of conventional autopsies. The first studies of the diagnostic value of postmortem radiology, indicates that postmortem radiology can be used as a replacement to an autopsy [16]. With this study we demonstrated that postmortem CT can be used for more than just imaging disease and cause of death; more precisely PMCT seems to be useful as a functional modality to estimate the postmortem interval.

The reasons for the postmortem increase in radiodensities are to be found in the normal postmortem changes of the body. It is already established from previous studies that electrolyte and biochemical parameters in CSF and VH increase with the PMI and show clear correlation to the PMI [7, 9, 17, 18]. This is probably caused by postmortem leakage through cell membranes and postmortem pleocytosis [19]. In a recent study, the influence of temperature on the radiodensities of body fluids was investigated and showed a minor influence; however, the fluids had been removed from the body and were heated or cooled. This process is not compatible with the natural physiological processes in the body after death [20]. Our results of intact cadavers showed no correlation with temperature in the multivariate model with a more natural decomposition process. The results of the 3 scan groups show a wide variation, which can partly be explained by differences in conditions and technique. The five donated cadavers were scanned in the same CT suite under standardized conditions of temperature and air humidity. The 100 in-hospital and 12 out-of-hospital cadavers showed a wider range of CSF and VH radiodensities, which can be explained by the differences in technique, environmental factors and condition of the body. As for technique, all scans in our study were performed with 120 kV. However, multiple CT scanners were used with slight differences in scanning parameters which can result in different radiodensity figures [21]. Environmental conditions during the PMI, such as clothing, the time period at room temperature or in the cooled mortuary setting, differed per cadaver and were not measured in this study. The donated cadavers all arrived in the CT suite without a cooling period, while the other cadavers may or may not have been stored in the cooled mortuary for an unknown period. Furthermore, a great diversity in the cause of death in our study population, with differing medication and treatment therapies, may have influenced the decomposition process and the radiodensities [4]. Future studies on the predictive value of PMCT for the PMI should investigate the more influencing factors such as body temperature and clothing.

Currently, the Henssge nomogram is the standard method for estimating the time of death. It makes use of the drop in body temperature after death compared to the ambient temperature, for example, wind and water temperatures and is corrected according to body weight and clothing. Other useful signs that are indicative of the time of death are rigor mortis, postmortem lividity and stages of putrefaction [5]. All of these tests are dependent on many environmental factors such as the ambient temperature. There are also influencing factors from within the body such as any pre-existing disease (for example, fever), medication usage, age, and body mass index. Therefore estimating the exact time of death is very complicated. It is not possible to give an exact time of death using the methods to calculate PMI, only an estimation within a time frame of a few hours. The estimation of time of death is useful in forensic settings and also in clinical postmortem investigations. It may be difficult for the radiologist to discern normal postmortem changes, from pre- or perimortal pathology, as these can look very similar [3, 4]. Calculating a fixed time or PMI on PMCT could aid the radiologist when determining normal postmortem physiology or pre-mortem pathology. For instance, the formation of gas in the liver is a normal postmortem finding that is more often found in cases of resuscitation, however, it can be a sign of pathology in the early postmortem hours in cadavers, such as bowel ischemia or infectious disease [22].

Other radiological methods for the PMI estimation have been investigated, such as MRI spectroscopy or PMMR diffusion in perinatal death, however these are labor-intensive and contrary to PMCT, PMMRI is often not available for postmortem scanning [23, 24].

We conclude that the radiodensities of VH and CSF are correlated to the PMI and can be helpful in estimating the time of death. Further studies are needed to evaluate the internal and external influencing factors as well as other potential characteristics on PMCT that can contribute to the estimation of the time of death.

## Key points

1. The radiodensities (Hounsfield units) of cerebrospinal fluid (CSF) and vitreous humor (VH) increase with the increasing postmortem interval (PMI).
2. A linear correlation is found between the CSF radiodensity and the PMI.
3. CSF radiodensity is a predictor of the PMI; and overrules body temperature in a multivariate regression model.
4. We present a formula to estimate the PMI using the radiodensity of CSF.
5. Postmortem computed tomography may be more than anatomical information; it may possibly be a helpful modality to estimate the PMI.
